# Analysis of Tensile Failure Behavior of Metal Fiber Laminates Under Different Temperature Environments

**DOI:** 10.3390/polym16233319

**Published:** 2024-11-27

**Authors:** Hongbin Lu, Dongfa Sheng, Yuting Fang, Hongquan Yu, Fan Yang

**Affiliations:** School of Civil Engineering, Southwest Forestry University, Kunming 650224, China; luhongbin1120@163.com (H.L.); fangyuting@swfu.edu.cn (Y.F.); 18227892325@163.com (H.Y.); fan_yang_study@163.com (F.Y.)

**Keywords:** fiber metal laminates, numerical simulation, temperature effects, tensile properties, microscopic damage, digital imaging correlation techniques

## Abstract

The tensile properties of fiber metal laminates were examined at temperatures ranging from 30 °C to 180 °C in this paper through the integration of numerical simulation techniques, experimental measurements, and digital image correlation techniques. The laminates were initially modeled using finite elements, and the failure behavior of porous basalt-fiber-reinforced aluminum alloy plates was numerically simulated. Consequently, metal fiber laminate stress–strain responses were varied by numerous tensile experiments conducted at varying temperatures. Simultaneously, a scanning electron microscope was used to scan a porous basalt-fiber-reinforced aluminum alloy laminate at different temperatures to determine the tensile mechanical behavior and micro-damage morphology. Lastly, the laminate’s dynamic response to the tensile process was observed through digital image correlation technology. The stress distribution was determined to be concentrated around circular openings through analysis. The strain distribution graph exhibited a “band” shape as the number of perforations increased. The findings indicate that fiber metal laminates lose tensile strength as temperatures increase. The ultimate tensile strength of the laminate decreases as the number of perforations increases at the same temperature. Complex damage mechanisms, including matrix debonding, fiber withdrawal, and matrix fracture, can be captured through scanning electron microscopy at varying temperatures. The tensile behavior and damage mechanisms of laminates with hole-containing structures under thermal conditions are examined, and the results can be used to inform the design and utilization of laminate structures.

## 1. Introduction

As a classic composite material, fiber metal laminate (FML) is made of thin sheets of alloy with a surface treatment and fiber-reinforced materials laminated alternately [1,2]. Based on different metals and fibers, FMLs have superior physical and chemical properties such as excellent specific strength, high specific stiffness, fatigue resistance and corrosion resistance [3,4]. These advantages give FMLs sustainable development prospects in aerospace, shipbuilding, the automotive industry, new energy and other fields. However, FML mechanical properties are sensitive to environmental conditions, especially the softening of the resin matrix material at high temperatures. This can lead to a decrease in the transverse tensile strength and bond strength between different layers [5,6]. Therefore, it is necessary to further investigate the tensile failure behavior of basalt-fiber-reinforced aluminum alloy laminates under varying temperatures and different pore numbers.

It is well known that FML mechanical behavior is particularly sensitive to high temperature conditions. With global temperatures rising, the mechanical properties of fiber-reinforced metal laminates at high temperatures have attracted widespread attention in recent years. Sun et al. [7] looked into the impact of temperature fluctuations from 30 °C to 160 °C on the tensile characteristics of polymers reinforced with carbon fiber, both theoretically and experimentally. The two failure modes were softening of the resin and delamination. In an experimental study, Cao et al. [8] investigated the tensile characteristics of hybrid laminates made of carbon-fiber-reinforced polymer, carbon/glass-fiber-reinforced polymer, and carbon/cobalt-fiber-reinforced polymer at temperatures ranging from 16 °C to 200 °C. The research findings indicate a considerable drop in the tensile strength of carbon fibers inside the same fiber-reinforced polymer sheet as temperature increases. Carbon-fiber-reinforced polymer composites may increase their tensile strength at high temperatures via fiber hybridization. Bhoominathan et al. [9] carbon-fiber-reinforced polymers were heated at 30, 60, 90, and 120 °C. The findings demonstrate that temperature and velocity have a major influence on material’s impact response. Ahmed et al. [10] examined the failure mechanisms of plastic materials reinforced with glass fiber at various temperatures and strain rates. Finding that fiber pull-out and fiber cracking predominated at high and low strain rates, respectively, contributes to a deeper comprehension of the failure process of glass-fiber-reinforced plastic composite materials. Ou et al. [11] investigated how temperature and strain rate affect the tensile characteristics and failure mode of unidirectional polymers reinforced with carbon fiber. The findings indicate that fiber debonding is more noticeable after failure and that temperature increases drop fiber bond strength. Yao et al. [12] investigated the tensile behavior of fiber metal laminates at various temperatures ranging from 25 °C to 175 °C. This was carried out by combining theoretical models, computational approaches, and experimental observations. According to the research, as the temperature rises, fiber metal laminates’ tensile strength reduces nonlinearly. Askari et al. [13] aged FML samples by thermal cycling and low-temperature cycling, and then performed impact tests to understand the effects of different aging methods on the mechanical properties of these structures. The experiment showed that during thermal aging, the impact strength tended to decrease due to the thermal degradation of the epoxy resin matrix when there were a high number of cycles or long exposure times. Multiple low-temperature cycles reduced the impact strength of FML. Cheng et al. [14] conducted impact tests on FMLs with different lamination sequences at temperatures of 25 °C, 30 °C, and 80 °C. The experimental results showed that the failure mechanism of FMLs at 30 °C changed significantly compared to 25 °C, and high temperatures of 80 °C had a significant effect on the failure of FMLs. Seoane-Rivero et al. [15] studied the mechanical properties of FML treated with NaOH at 175 °C. The study showed a significant improvement in the tensile strength of FML.

Fiber metal laminates shrink depending on temperature. In addition to the aforementioned tests, the porous structure of laminates is also widely used in technical applications. Studies on the failure behavior and residual strength of porous structures are also supported by the literature. Jiang et al. [16] studied variations in residual strength, damage evolution process, damage influence area, and geometric features of holes in fiber metal laminates. It was found that square holes damage fibers more than circular ones. The residual strength of glass fiber-reinforced vinyl ester laminates with numerous holes was investigated by Kazemahvazi et al. [17]. The authors developed an analytical model for damage residual strength useful for evaluating laminate mechanical characteristics. A finite element simulation model was presented by Gerendt et al. [18] in order to forecast the progressive damage failure manner of bolt connections laminated with metal fiber. The failure behavior of fiber metal laminated bolt connections is correctly predicted by numerical simulation, which also fits well with the experimental data. The tensile mechanical behavior and progressive damage morphology of laminates made of magnesium alloy reinforced with glass fiber and varying hole counts were investigated by Lin et al. [19]. It was found that when the number of holes increases, the final tensile strength decreases. Few studies have been carried out on porous structures; the majority of the aforementioned studies have been on single-hole FMLs. In addition, most studies on the tensile characteristics of porous FML have been conducted at room temperature. Few studies have been conducted on the physical properties and residual power of porous materials at varying temperatures. The use of porous structures in the transportation sector is growing as a result of global scientific and technological advancements, necessitating more research into the tensile failure behavior of porous FMLs.

Due to the widespread use of FMLs in engineering constructions, a detailed investigation of their characteristics has been conducted via analytical models, computational approaches, and experimental methods. There are several types of properties, including fatigue resistance, impact resistance, knock resistance, and flexural resistance. Researchers have also investigated their tensile qualities. Cortes et al. [20] examined the impact energy absorption characteristics and tensile strength of magnesium composite laminates reinforced with fibers. The findings indicated that the tensile strength of the FML fiber layer can be substantially enhanced by increasing its volume percentage. In addition, the fiber layer can significantly improve fatigue resistance. The laminate’s impact energy absorption capacity can be significantly improved by the delamination and shear fracture in the magnesium alloy layer. These phenomena have been confirmed in additional experimental studies [21,22]. Moussavi-Torshizi et al. [23] used statistical analysis to estimate the ultimate tensile strength of FMLs in relation to the fiber direction. Furthermore, FML tensile properties have been extensively investigated in relation to other parameters, such as hygrothermal conditions [24,25], strain rate [26], and notch shape [27,28]. Nevertheless, the majority of the aforementioned investigations were conducted at ambient temperature. Only Sarasini et al. [22] conducted an experimental investigation into the impact behavior of thermoplastic fiber metal laminates in response to different temperatures. The superior deformation capacity of TFMLs at low temperatures (−40 °C) is the reason for the higher specific energy required to initiate the first fracture in TFMLs than in other structures, they concluded. In a three-point bending test, Kubit et al. [29] examined the impact of thermal stress on the FML mechanism. Thermal shock cycles were discovered to be closely associated with interlaminar shear strength and failure mode. It is evident that FML’s mechanical properties and failure behavior are significantly influenced by temperature. Nevertheless, there is a dearth of information regarding the impact of temperature on FML tensile properties, particularly at high temperatures.

The tensile damage and damage mechanism of porous basalt-fiber-reinforced aluminum alloy laminates at varying temperatures (30–180 °C) are examined in this paper through data simulation, experimental characterization, and digital imaging techniques. Porous laminates were subjected to tensile experiments at varying temperatures. Digital imaging techniques also observed laminate tensile failure behavior. In order to characterize the microscopic damage patterns at the fracture of laminates at various temperatures, including matrix damage, fiber fracturing, fiber withdrawal, and interlayer delamination, scanning electron microscopy (SEM) was employed. Subsequently, this paper offers an exceptional reference for a variety of porous designs and applications of basalt-fiber-reinforced aluminum alloy laminates under room-temperature conditions, utilizing digital imaging techniques and experimental data. Furthermore, numerical simulations of damage progression were implemented for aluminum alloy, basalt fibers, and binder layers. The tensile response and degradation modes of laminates under room-temperature conditions were predicted using these simulations.

## 2. Preparation of Samples and Experimental Development

### 2.1. FML Preparation

In this study, basalt fibers and epoxy resin were processed before use. The composite layer of FML was basalt/epoxy resin (BF/EP) consisting of basalt fibers selected by full impregnation with epoxy resin and hardener. Basalt fiber prepregs were obtained from Haining City (Dingqiao Anbang Building Materials Department, China), and the basalt/epoxy resin was oriented according to [0°/90°] in the test. The FML samples were prepared by hand lay-up according to [Al/0°/90°/Al/90°/0°/Al]. The unidirectional basalt/epoxy layer thickness was 0.21 mm, and its mechanical property parameters are shown in Table 1.

The aluminum alloy is 2024-T3 with a thickness of 0.5 mm, which is stretched to obtain isotropic hardening data (engineering stress and engineering strain). The mechanical properties and parameters of 2024-T3 aluminum alloy are shown in Table 2.

**Figure 1:**
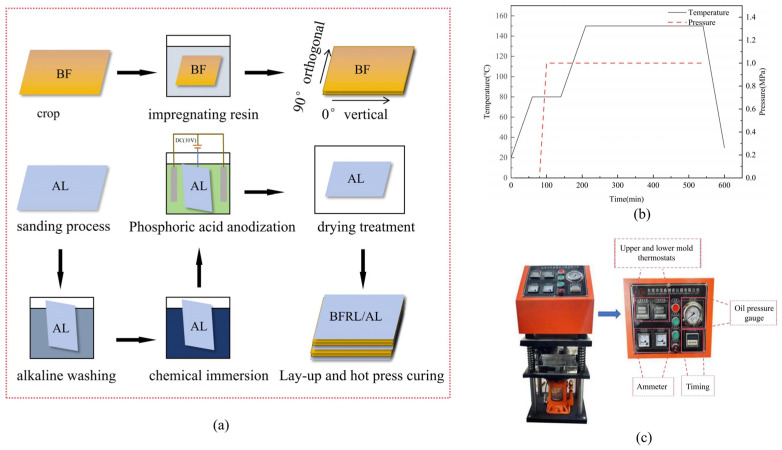
Diagram of the BFRL/AL forming process. (**a**) Experimental specimen production, (**b**) heat-pressing process, (**c**) hot pressure test machine. (BF: Basalt fiber; DC: Direct current).

The tensile and mechanical properties of laminates are affected by problems with interlayer bonding between the basalt/epoxy resin layer and aluminum alloy layer. In order to strengthen the bonding between the fiber and the aluminum alloy layer, the preparation process of the laminate was improved. The surface of the aluminum alloy layer was firstly sanded using silicon carbide sandpaper and angle grinder, and the surface was cleaned with acetone, followed by alkaline washing and deoxidation of the surface of the aluminum alloy, phosphate anodization of the aluminum alloy after rinsing, and finally, the aluminum alloy was put into the DHG-9030A blower drying oven for drying. A layer of interface resin and curing agent was brushed uniformly on the sanded surface of the aluminum alloy layer and the surface of prepreg basalt fiber, and the layers were laid up according to the lay-up sequence [Al/0°/90°/Al/90°/0°/Al], and then put into the hot molding curing equipment for curing. Figure 1 shows the laminate preparation process details.

Figure 1b,c shows the details and parameters of the FML heat-pressing curing machine. At the beginning of the temperature rise, equipment thermos-molding layer preheating needs to be carried out; when it reaches 80 °C, pressurized thermal curing occurs on the lay-up good specimen, by first setting the pressure of 1.0 MPa on the plywood pressurization for 30 min, followed by a rise in the temperature to reach 150 °C to continue to hot press for 240 min, from which it is removed and cooled to room temperature.

**Figure 2:**
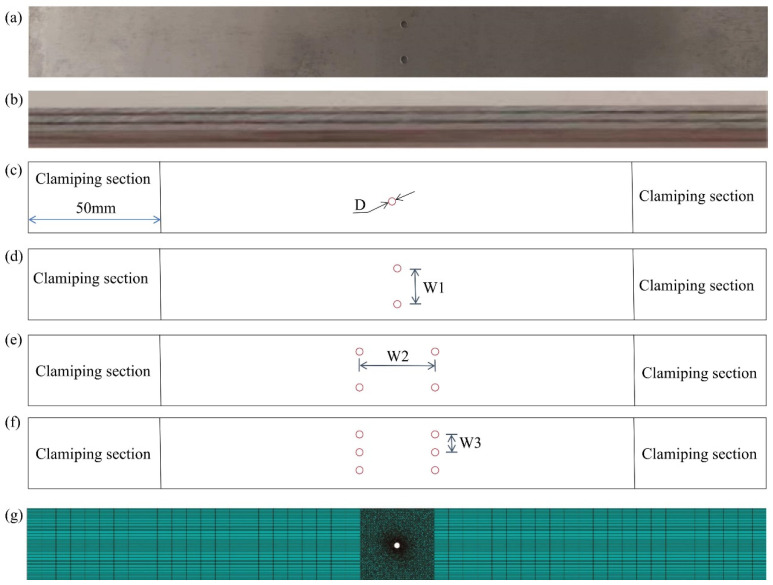
FML specimen information in detail. (D: diameter). (**a**) Test sample, (**b**) sample side, (**c**–**f**) sample dimension, (**g**) numerical analysis model of the sample.

In this study, the FMLs were completed by heated compression curing and then cut, punched, and drilled using a metal machine. This was performed to generate FML samples with varying spacings and perforation counts, as illustrated in Figure 2. The samples were 250 mm in length and 25 mm in width, and their parameters conformed with the ASTM-D3039 standard. The aperture spacing was w_1_ = 12.5 mm, w_2_ = 25 mm, and w_3_ = 6.25 mm, and the clamping length was 50 mm. Figure 2c–f illustrates this. Ultimately, the prototypes were refined.

### 2.2. Methods of Experimental Investigation

#### 2.2.1. Tensile Test Experiment

**Figure 3:**
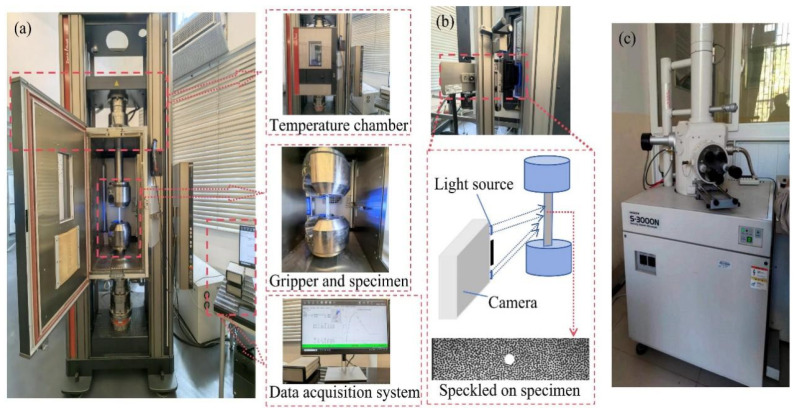
Tensile test for various temperatures. (**a**) Zwick/Roell tensile test machine, (**b**) DIC test machine, (**c**) SEM test of fracture specimens.

Quasi-static tensile properties were measured at a loading rate of 2 mm/min using a Germany Z250 universal electronic tensile testing machine in this study. The Zwick/Roell tensile testing machine (Ulm, Germany), with a load capacity of 250 KN, is illustrated in Figure 3a. The sample was centered within the fixture. A temperature chamber was included in the tensile tester to enable experiments to be conducted at a specific temperature. The specimens’ tensile properties were evaluated in a heated compartment at temperatures spanning from 30 °C to 180 °C, as illustrated in Figure 3a. Thermal stability was guaranteed by a 5 °C/min heating rate. This study examined the mechanical property changes and damage conditions at high temperatures as a consequence of the temperature chamber simulating the potential operating ambient temperature of FML.

#### 2.2.2. Digital Imaging Correlation Technology (DIC)

The digital image assessment system depicted in Figure 3b is composed of a high-precision camera, two special light sources, a supporter, a high-performance computer, and XTDIC Ver. 9.5 software. Digital image correlation algorithms match deformation points on the object surface. A high-precision camera captures dispersed images of the sample at each deformation stage using a digital image testing system. The 3D coordinates of the calculated points on the object’s surface are reconstructed based on the parallax data of each point. The strain field of the specimen is calculated by comparing the three-dimensional coordinate changes of each point in the measurement area of each deformation state to the displacement field of the specimen’s surface. Digital image examination is conducted concurrently with the tensile test.

The laminate damage pattern after fracture was further observed. A small specimen was obtained by removing the fractured specimen at the end of the tensile experiment. It was cut at a distance of 5 mm from the fracture. The small specimen was fixed to a balance table with carbon conductive tape. Air was extracted from it after putting the balance table into the particle-sputtering device. Gold atoms were sputtered onto the specimen under a vacuum, increasing its visibility under an electron microscope. Using an electron microscope, the specimen’s failure mode was observed according to standard operating procedures. This is shown in Figure 3c.

## 3. Finite Element Modeling and Damage Criteria

Abaqus ACE 2021 was used to conduct a finite element analysis of laminate under tension in this study. In order to numerically simulate the damage modes and behaviors of the aluminum alloy layer, the fiber layer, the resin layer, and the bonded layer, this was carried out. Subsequently, the VUMAT subroutine was modified to incorporate the metal damage model, the fiber failure model, and the interlayer delamination model. This was employed to specify the progressive damage modes of each stratum.

### 3.1. Metal Damage Modeling

The Johnson–Cook model takes into account strain, stress triaxiality, and temperature, and evaluates the effects of damage to metallic materials under complex stress, deformation, and temperature; so, the Johnson–Cook model is widely used for metallic materials damage failure models. The Johnson–Cook intrinsic model is used for aluminum plates with the following expression:

(1) $$\sigma =\left[A+B\;\varepsilon^{n}\right]\left[1+C\;\ln\varepsilon^{*}\right]\left[1-{\left(T^{*}\right)}^{m}\right]$$

where *A* is the quasi-static yield strength, *B* is the strain intensification factor, *C* is the strain rate sensitivity factor, *n* is the strain hardening factor, *m* is the temperature sensitivity factor, and ε is the strain and *T* is the temperature.

The Johnson–Cook model proposed a definition of material damage for aluminum alloy damage evolution:

(2) $$D=\sum \frac{\Delta \varepsilon }{\varepsilon_{f}}$$

where *D* is the damage parameter. When *D* = 1, the material breaks. Δε is the increment in equivalent plastic strain; and $\varepsilon_{f}$ is the fracture equivalent strain. The Johnson–Cook failure model that simultaneously takes into account the model strain, stress triaxiality, and temperature, the failure model defines the equivalent fracture strain as follows:

(3) $$\varepsilon_{f}=[(D_{1}+D_{2}\exp(D_{3}\sigma^{*})][1+D_{4}\ln(\overset{\cdot }{\varepsilon })](1+D_{5}T^{*})$$

where $\sigma^{*}=p/\sigma_{\mathrm{eff}}$, *p* is the pressure, $\sigma_{\mathrm{eff}}$ is the equivalent force, ${\dot{\varepsilon }}^{*}=\dot{\varepsilon }/{\dot{\varepsilon }}_{0}$ is the relative equivalent plastic strain rate, $\dot{\varepsilon }$ is the plastic strain rate, ${\dot{\varepsilon }}_{0}$ is the reference plastic strain rate, which can be taken as ${\dot{\varepsilon }}_{0}=1\;s^{-1}$; $T^{*}=(T-T_{r})/(T_{m}-T_{r})$ is the dimensionless temperature, and $T_{r}$, $T_{m}$ are the melting point and the room temperature, respectively; *D*_1_, *D*_2_, *D*_3_, *D*_4_, *D*_5_ is the damage parameter (see Table 3 [30]).

### 3.2. Fiber Failure Model

Currently, commonly used criteria for predicting laminate failure include the Tsai-Wu criterion [31], the Chang criterion [32] and the Hashin criterion [33]. The Hashin criterion and Yeh delamination criterion [34] are used for laminate failure analysis because they facilitate the determination of the failure mode. The Hashin criterion considers four failure modes: fiber tensile failure, fiber compressive failure, matrix tensile failure and matrix compressive failure. The Yeh delamination criterion considers delamination failure. The five failure criteria are as follows:

Fiber Stretch Failure $\left(\sigma_{11}>0\right)$:

(4) $${\left(\frac{\sigma_{11}}{X_{T}}\right)}^{2}+{\left(\frac{\tau_{12}}{S_{12}}\right)}^{2}+{\left(\frac{\tau_{13}}{S_{13}}\right)}^{2}\geq 1$$

Fiber Compression Failure $\left(\sigma_{11}<0\right)$:

(5) $${\left(\frac{\sigma_{11}}{X_{c}}\right)}^{2}\geq 1$$

Matrix Tensile Failure $\left(\sigma_{22}+\sigma_{33}>0\right)$:

(6) $${\left(\frac{\sigma_{22}+\sigma_{33}}{Y_{T}}\right)}^{2}+\frac{1}{S_{23}^{2}}\left(\tau_{23}^{2}-\sigma_{22}\sigma_{23}\right)+{\left(\frac{\tau_{12}}{S_{12}}\right)}^{2}+{\left(\frac{\tau_{13}}{S_{13}}\right)}^{2}\geq 1$$

Matrix Compression Failure $(\sigma_{22}+\sigma_{33}<0)$:

(7) $$\begin{array}{l} \left(\frac{\sigma_{22}+\sigma_{33}}{Y_{T}}\right)\left[{\left(\frac{Y_{c}}{2S_{23}}\right)}^{2}-1\right]+\frac{{\left(\sigma_{22}+\sigma_{33}\right)}^{2}}{4S_{23}}+\\ \frac{1}{S_{23}^{2}}\left(\tau_{23}^{2}-\sigma_{22}\sigma_{33}\right)+{\left(\frac{\tau_{12}}{S_{12}}\right)}^{2}+{\left(\frac{\tau_{13}}{S_{13}}\right)}^{2}\geq 1 \end{array}$$

Layering Failure $(\sigma_{33}>0)$:

(8) $${\left(\frac{\sigma_{33}}{Z_{T}}\right)}^{2}+{\left(\frac{\tau_{13}}{S_{13}}\right)}^{2}+{\left(\frac{\tau_{23}}{S_{23}}\right)}^{2}\geq 1$$

where $\sigma_{ij}$ is the corresponding surface positive stress on the metal plywood; $\tau_{ij}$ is the shear stress on the metal plywood $X_{T}$, $Y_{T}$, $Z_{T}$ for the unidirectional metal plywood ply in each direction tensile strength; $X_{c}$, $Y_{c}$ for the unidirectional metal plywood ply in each direction compressive strength; and $S_{\mathrm{ij}}$ for the direction of the face within the shear strength.

### 3.3. Hierarchical Model

A bilinear adhesion zone is the determining factor for bonding interface damage between laminates. The traction–detachment law can be used to define the adhesive unit’s elasticity as follows [35]:

(9) $$\left[\begin{array}{l} \sigma_{n}\\ \sigma_{s}\\ \sigma_{t} \end{array}\right]=\left[\begin{array}{ccc} \begin{array}{l} E_{\mathrm{nn}}\\ 0\\ 0 \end{array} & \begin{array}{l} 0\\ E_{\mathrm{ss}}\\ 0 \end{array} & \begin{array}{l} 0\\ 0\\ E_{\mathrm{tt}} \end{array} \end{array}\right]\left[\begin{array}{l} \varepsilon_{n}\\ \varepsilon_{s}\\ \varepsilon_{t} \end{array}\right]$$

Degradation begins when the stresses or strains satisfy the defined initial critical damage criterion. In this paper, the Quads criterion in ABAQUS is utilized and publicized as follows [19]:

(10) $${\left\{\frac{\sigma_{n}}{\sigma_{n}^{0}}\right\}}^{2}+{\left\{\frac{\sigma_{s}}{\sigma_{s}^{0}}\right\}}^{2}+{\left\{\frac{\sigma_{t}}{\sigma_{t}^{0}}\right\}}^{2}=1$$

where $\sigma_{n}$ is the nominal stress under the principal stress, $\sigma_{s}$ is the nominal stress in the first shear direction, and $\sigma_{t}$ is the nominal stress in the second shear direction.

The Benzeggagh–Kenane damage criterion [36] is used to determine the initiation of damage:

(11) $$G_{\mathrm{IC}}+\left(G_{\mathrm{IIC}}-G_{\mathrm{IC}}\right){\left(\frac{G_{\mathrm{II}}+G_{\mathrm{III}}}{G_{I}+G_{\mathrm{II}}+G_{\mathrm{III}}}\right)}^{\eta }=G_{C}$$

where $G_{\mathrm{IC}},G_{\mathrm{IIC}},G_{\mathrm{IIIC}}$ denotes the dissipation energy of the viscous elements in different directions, respectively, and *C* denotes the critical fracture energy. The cohesive layer properties are shown in Table 4.

## 4. Finite Element Modeling

The finite element method was used to model the response of FML under tension. In the modeling process, the layers of material in FML (aluminum alloy layer, basalt fiber layer, resin layer and bonding layer) are uniformly distributed on a microscopic scale, and their material properties do not change with the position within the respective layers. At the same time, it is assumed that the bonding between the layers is ideal; that is, in the initial state, there are no defects or gaps between the layers, and the bonding layer can uniformly transfer stress and strain. Finally, it is assumed that the stress–strain relationship of the FML follows linear elastic law during the elastic deformation stage. The metal and fiber layers are discretized using eight-node linear hexahedral elements (C3D8R), and the bonding layer uses eight-node three-dimensional cohesive elements (COH3D8). The mesh size near the opening is 0.5 mm × 0.5 mm to accurately capture stress concentration and damage evolution. Away from the tensile area, the mesh gradually becomes coarser, and the mesh size can be increased to 2–5 mm, which not only ensures calculation accuracy in key areas, but also reduces the overall calculation amount, as shown in Figure 2g. A fixed constraint is applied at one end of the model to restrict the translational and rotational degrees of freedom of all nodes at this end to simulate the situation where one end of the test piece is fixed in an actual tensile test. A displacement load in the tensile direction is applied to the nodes at the other end to simulate the loading process in the tensile test. The displacement loading rate is 2 mm/min, which is consistent with the experimental loading rate, so as to ensure the comparability of the simulation and experimental conditions. When simulating the tensile mode, the mesh deletion of the component is defined by using the cross-section control in Abaqus, that is, if failure degradation is reached during the damage evolution process, the mesh will be deleted.

### 4.1. Examination of Finite Element Simulation Results

The fiber metal laminate comprises the fiber layer, matrix layer, and a portion of the metal layer, as a laminated composite construction. Consequently, the process of laminated fractures deteriorating is dynamic. In a microcosm, it is challenging to observe the gradual expansion of harm in each material layer. Nevertheless, the finite element method (FEM) enables an easy observation of damage changes and fracture extension in each material layer. Finite element simulation analysis is employed to investigate the deformation process and failure behavior of the laminate in this investigation. In order to gain a more comprehensive understanding of FML failure behavior, this section contrasts the tensile testing data with the FEM modeling results of four specimens with varying apertures at room temperature (30 °C). Laminates are extensively acknowledged for their intricate architectures. Using the progressive damage model of the aluminum alloy plate, bonding layer, and composite layer, this study examines the progressive failure behavior of FMLs over time.

**Figure 4:**
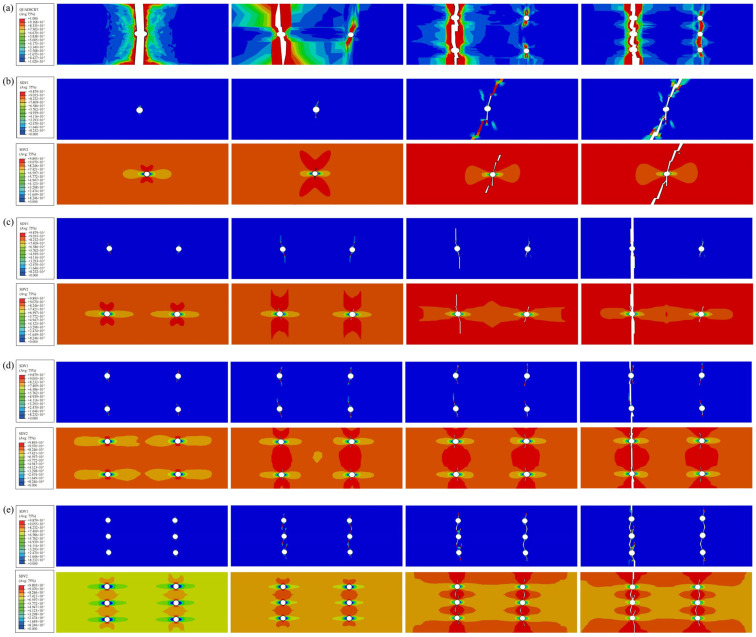
Damage evolution of composite layers. (**a**) Comparison of interlayer damage between FML specimens, (**b**) single hole, (**c**) two holes, (**d**) four holes, (**e**) six holes.

The bond failure between the metal layer and the fiber layer is depicted in the QUADSCRT plot. The plot illustrates the bond’s complete failure in red. Figure 4a illustrates the bond damage region for varying perforations numbers. While the burden increases, the delamination damage region extends from the perforations’ perimeter to its two extremities. Ultimately, the region exhibits a “funnel” form. The bond’s injury diminishes as the number of fractures increases. Consequently, the FML’s rigidity decreases as the number of perforations increases at the same temperature.

SDV is the state variable of the fiber or matrix layer of the finite element software calculation program, where SDV1 and SDV2 are the fiber layer tensile damage state and matrix layer tensile damage state, respectively. The blue area in the FEM represents 0° fiber ply damage, orange represents 90° matrix ply damage, and the white area indicates complete unit deletion. The unit is deleted when the damage state reaches 1 failure, and it is no longer capable of bearing or transmitting burdens. The progressive damage simulation of the fiber layer (SDV1) and matrix layer (SDV2) of BFRL/AL plywood is depicted in Figure 4b–e.

As the load increases, the stress is concentrated in the region above the circular hole. This is the first region to experience damage. The fiber layer cracks extend longitudinally along the circular hole. The damage zone of the matrix layer gradually expands from the original “butterfly shape” to “hourglass shape”. The matrix layer is the first to damage, and the 0° fiber layer mainly carries the matrix; with the 0° fiber matrix layer in the load gradually increasing, the damage area expands the formation of hourglass shape, finally leading to matrix failure. In the process of matrix damage, the 90° fiber layer mainly prevents the damage evolution of the matrix layer. However, when it reaches a certain load, the 90° fiber perforates longitudinally, and finally fiber and matrix damage failure occurs. The more holes there are, the larger the white area (indicating unit deletion) and the smaller the effective bearing volume, resulting in lower residual strength.

### 4.2. Comparison of Finite Element Simulation Results with Experimental Results

**Figure 5:**
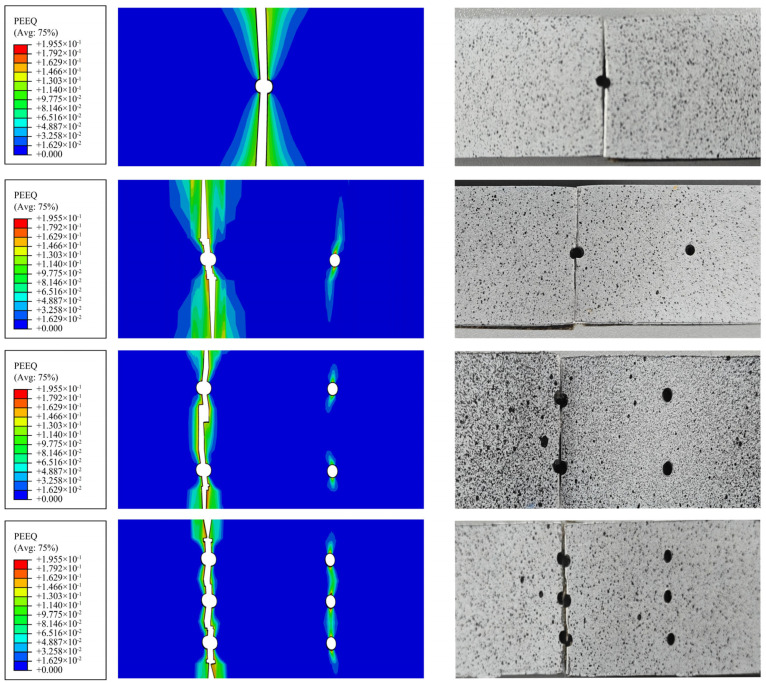
Comparison of equivalent plastic strain damage in the tension of FML specimens with holes.

The fracture mode of the metal layer simulated by the Johnson–Cook damage model is essentially identical to that of the tensile test. Stress concentration is observed in the vicinity of the circular cavity, as indicated by finite element analysis. Some degree of delamination and brittle fracture is present with the fracture mode. It is evident that the plastic injury area of the metal layer increases as the number of analysis stages and the burden increase. Plastic yielding and necking are also evident in the aluminum alloy layer cross-section. The observed range of fracture failure is consistent with the expansion of cumulative plastic deformation in the aluminum alloy layer. This is demonstrated through a comparison of the experimental strain diagram and the PEEQ equivalent plastic strain diagram, as illustrated in Figure 5.

## 5. Analysis of Experimental Results

### 5.1. Tensile Response Analysis

**Figure 6:**
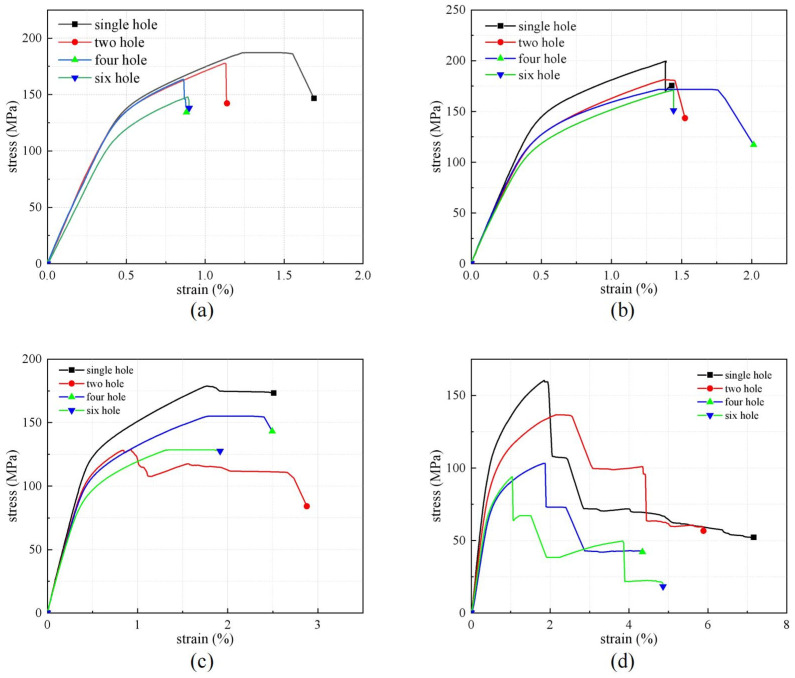
Stress–strain curves and mechanical responses of FML residual strength with various openings. (**a**) 30 °C, (**b**) 80 °C, (**c**) 130 °C (**d**) 180 °C.

Multiple samples were tested in each temperature range according to the approach described in Section 2.2. The present paper examines tensile tests performed at temperatures ranging from 30 to 180 °C, and the data were acquired using a data acquisition system. The stress–strain curves of FMLs at different temperatures have enough similarity to ensure experimental data reliability. From Figure 6a, the FML stretching of single holes at a temperature of 30 °C took place through four classical stages: stages of elasticity, super-yield, post-yield, and load-bearing damage. Due to the ply orientation of the fiber layers of the FMLs at 0°/90°, these tensile stress–strain curves shown in Figure 6a,b decrease abruptly at the final stage, and FML fracture occurs. However, in the case of 180 °C, as shown in Figure 6d, a big difference occurs. The amount of stress decreases abruptly after ultimate tensile loading. The curve shows a stepwise decrease with strain increase, and finally the stress remains steady. This phenomenon is attributed to the softening of the matrix due to the increase in temperature. This is related to the limiting temperature of the epoxy resin adhesive (around 170 °C). As a result, interlayer peeling occurs between the metal layer and the composite laminate. The maximum stress indicates the ultimate stress at which the aluminum alloy layer on the surface of the composite laminate will eventually fracture. As shown in Figure 6, at the same temperature, with the increase in the number of holes, the bearing area of the FML decreases, and the critical damage range at the edge of the laminate holes expands, resulting in the porous laminate being smaller than the single-hole laminate, and the average bearing capacity decreases by 32.63%. As the temperature increases from 80–180 °C, the delamination of laminate becomes more serious.

**Figure 7:**
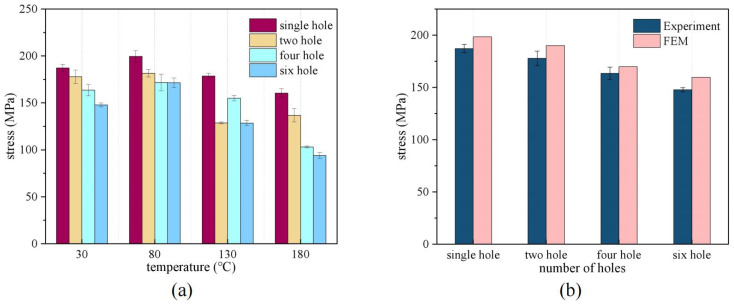
Comparison of ultimate stresses. (**a**) Residual stresses of FMLs at different temperatures, (**b**) finite element and experimental residual stress comparisons.

Based on the stress–strain curves in Figure 6, Table 5 records the ultimate tensile stresses of the same specimen for three experiments at the same temperature. In addition, the average value for each temperature case was calculated. In order to make a more intuitive comparison, ultimate tensile stresses at different temperatures with varying numbers of holes were plotted according to this table, as shown in Figure 7a. For the tensile load versus temperature of FML, it can be found that the strength of specimens with single holes decreased less at different temperatures. This was with an average decrease of 10.72% at 180 °C equivalent to 30 °C. As a whole, the tensile load decreases at temperatures above 80 °C due to localized delamination caused by matrix softening. The results show that the ultimate tensile stress at 180 °C is drastically reduced (33.9%) compared to the 130 °C condition. An interesting phenomenon worth noting is the slight increase in tensile stress at 80 °C (6.23%) compared to that at 30 °C. This may be related to the curing process, in which the temperature of the specimen preparation process facilitates the elimination of curing defects in the resin matrix. In addition, the temperature increase effectively eliminates internal stresses generated during hot pressing. Therefore, the elimination of defects in the epoxy resin matrix at higher temperatures improved the specimens’ tensile characteristics.

Furthermore, Figure 7b illustrates the comparison between the finite element and experimental residual strengths of the specimens. The experimental and finite element results exhibit residual strength differences of 5.99%, 6.93%, 3.85%, and 7.98%, with an average difference of 6.19%. Consequently, the numerical simulation results are in substantial agreement with the measured data and are informative for the prediction of FML structural strength. Lastly, the FML’s prediction model is an optimal structure due to specific structural defects during processing. Consequently, it is consistent with other studies that the FMLs predictions are greater than the measured measurements.

### 5.2. Tensile Damage Mechanism

**Figure 8:**
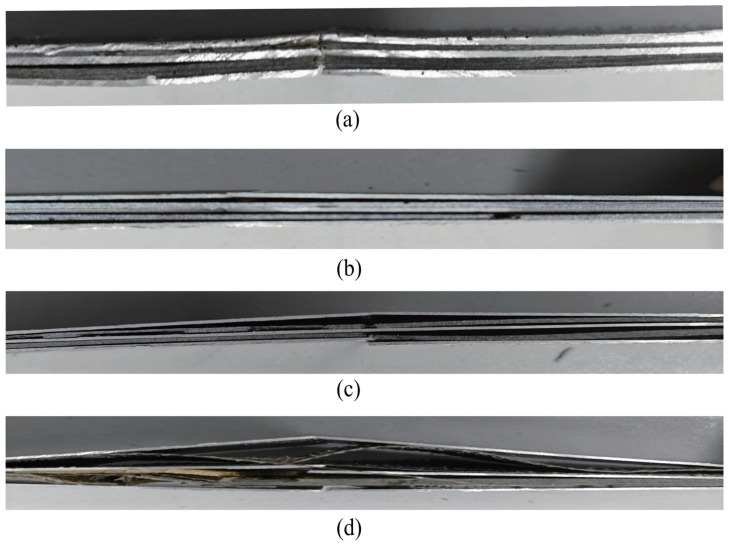
Specimens with a single-hole specimen side surface following a tensile test. (**a**) 30 °C, (**b**) 80 °C, (**c**) 130 °C, (**d**) 180 °C.

The delamination phenomenon between the fiber layer and the aluminum alloy layer is evident in Figure 8a following the damage. The initial damage to the fiber and matrix was caused by the change in stress gradient between the fiber layer and the aluminum alloy layer. This was during the FML tensile process. As a consequence, the toughness damage expanded rapidly along the crack direction, resulting in a reduction in the load-bearing capacity. Subsequently, a delamination phenomenon occurred between the FML layers, and the final damage to the FML as a whole occurred. The fiber layer was subjected to the majority of the load when the temperature was between 30 °C and 130 °C, and it fractured prior to the aluminum alloy layer during the tensile process. This was a contributing factor to the FML’s load-bearing capacity loss, as it resulted in a rapid reduction in the ultimate load-bearing capacity.

The specimen fracture is relatively planar at the fracture site when the temperature is lower than 130 °C, as illustrated in Figure 8. A modest delamination phenomenon occurs between the layers. The delamination damage becomes more severe as the temperature exceeds 130 °C, and the fracture extends to both extremities. The fractured specimen demonstrates decoupling of the resin from the matrix in the fiber layer. The bonding layer between the fiber and the metal layer exhibits a clear damage phenomenon as the temperature and tensile load increase. It is also possible to infer that the matrix’s softening becomes more pronounced when the environment’s temperature surpasses the melting temperature. Between the strata, a substantial quantity of resin matrix fragments is observed. The fiber layer in the specimen did not fracture when the test temperature was increased to 180 °C. There was only one apparent fissure in the metal layer, and resin matrix fragments were visible between the layers. Therefore, the aluminum alloy layer assumed the primary load-bearing function when the temperature exceeded 170 °C.

### 5.3. Microscopic Damage Mechanism at the Fracture Site

The use of scanning electron microscopy (SEM) enables a more comprehensive understanding of the microscopic aspects of damage patterns in FMLs, as some of the damage is not readily apparent due to the complexity of the damage.

**Figure 9:**
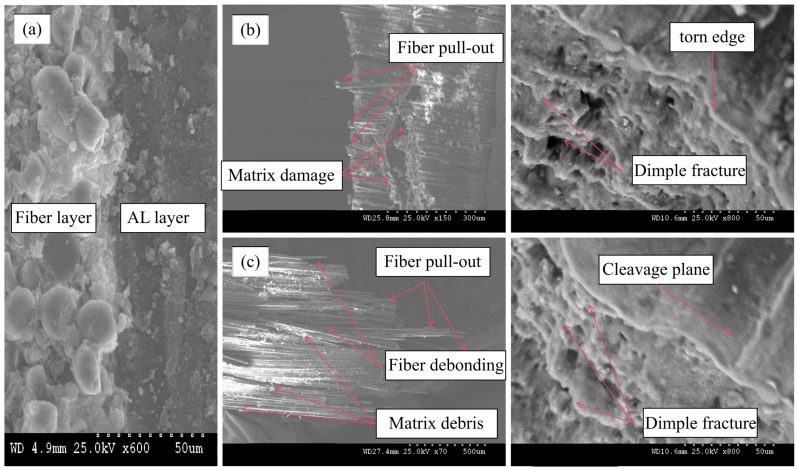
Microscopic diagram of FMLs fracture surface. (**a**) Side microscopic graph, (**b**) 30 °C, (**c**) 180 °C.

As can be seen in Figure 9a, the transverse microscopic morphology of the surface-treated FMLs has better interlayer bonding integrity, which effectively reduces the defects such as interlayer delamination, mislayer, and microholes, and improves the compactness of the specimen interlayers. Figure 9b shows the microscopic damage morphology of the FML fracture at 30 °C. Many obvious damage patterns, including fiber pullout and matrix cracking, can be clearly captured from SEM images. In addition, smooth fiber breakage can be observed in the fracture region due to the tensile loading direction coinciding with the lay-up direction. In order to find the obvious changes in damage patterns at high temperatures, the fine-scale damage of FMLs at 180 °C is shown in Figure 9c, where the FMLs show different damage patterns at fracture. The fiber layer fracture zone is more fragmented due to more matrix fractures. The debonding of the fibers from the matrix is also clearly visible. The fracture region shows a lot of matrix debris and fiber pull-out. All of these phenomena are due to an increase in tensile loading that destroys the matrix structure, resulting in matrix cracking, fiber debonding and pull-out. In addition, more importantly, the resin softens more and more obviously as the temperature increases. This weakens the bonding strength between the fiber layer and the matrix. This leads to fiber pull-out and ultimately to a reduction in the fiber bundle’s tensile strength.

The analysis of the fracture micro-morphology of the metal layer shows that the aluminum alloy layer has obvious layer fracture characteristics with edge-tearing after tensile fracture, in addition to the fracture surface showing corrugation and raised nodules. When the tensile load increases, the deformation of the aluminum alloy also increases. The walls of the micropores inside the metal continue to thin, and eventually tear to form many toughness fractures. It can be concluded that the FML damage destruction mode is a mixture of multiple damages.

### 5.4. Digital Image Correlation Analysis

**Figure 10:**
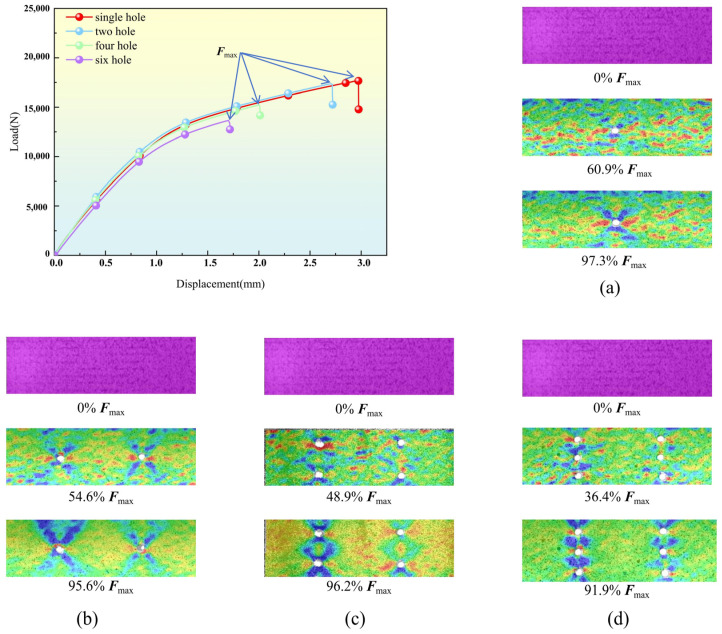
The DIC technique was used to derive strain fields for specimens under various openings. (**a**) single hole, (**b**) two holes, (**c**) four holes, (**d**) six holes.

The DIC method was employed to measure the surface strain under various pressures to further observe the tensile response of plywood with varying pore layers at room temperature, as illustrated in Figure 10. The strain contours of the load–displacement curve for ‘one hole’, ‘two holes’, ‘four holes’, and ‘six holes’ are depicted in each sub-figure of Figure 10. The origin (no loading) and the other two loading sites (shown at the bottom of the sub-figure) are represented. For each sample with varying perforation numbers, the strain distribution was determined at three loading points. The strain evolution of a specimen with a single perforation at ambient temperature (30 °C) is illustrated in Figure 10a. The initial phase is relatively consistent. The tension distribution is concentrated near the circular opening as the burden increases. The strain distribution diagram exhibits a ‘butterfly’ shape until a fracture occurs. It is critical to note that the tensile strain field is not symmetrically distributed at 95.6% Fmax (Fmax is the peak load at room temperature), as shown in Figure 10b. This may be the result of random defects in the specimen and potential eccentric loading. The tension distribution becomes more concentrated and adopts a “double X” configuration when the number of perforations reaches four, as illustrated in Figure 10c. Nevertheless, as illustrated in Figure 10d, the load-bearing capacity abruptly decreases when the number of openings is increased to six. The strain distribution is characterized by a “banded” structure, as the stress distribution is concentrated around the circular opening. As a result, the maximum load-bearing capacity of the specimen is reduced as the number of perforations increases.

## 6. Conclusions

The tensile behavior and damage mechanism of FMLs at various temperatures are primarily examined in this paper through numerical simulation, experimentation, and digital imaging techniques. FML’s tensile properties are thoroughly examined in relation to perforations. First, a finite element model was created, and the numerical analysis of the tensile strength and progressive damage evolution process of FMLs was conducted at room temperature. Then, a variety of tensile experiments were performed on FMLs at varying temperatures. In the interim, scanning electron microscopy was employed to characterize the microscopic damage mechanism of FML fractures. Ultimately, the tensile dynamic response was demonstrated through digital imaging techniques. Several noteworthy conclusions can be drawn from integrating these diverse investigation methods. This results in a reduction in the specimen’s maximal load-bearing capacity.

(1) The tensile strength and failure modes were essentially the same at temperatures ranging from 30 °C to 80 °C.

(2) The tensile load of basalt-fiber-reinforced aluminum alloy laminate specimens exhibits clear nonlinearity and decreases as temperature increases. The specimen’s residual strength decreases and fracture strain increases when the working temperature surpasses 130 °C. This is the result of interlayer delamination degradation and resin matrix softening. Consequently, it is not advisable for the FML’s working temperature to exceed 130 °C.

(3) The numerical simulation results of basalt-fiber-reinforced aluminum alloy laminates were compared to the experimental results, and the results were determined to be consistent with the experimental mechanical response and damage patterns. In comparison to standard room-temperature conditions, the residual strength values of the specimens demonstrate an average difference of 6.19%. The residual strength diminishes as the number of perforations increases at the same temperature.

(4) The failure mode of FMLs is closely related to temperature conditions. Scanning electron microscopy analysis revealed that matrix cracking, fiber pullout, and fiber matrix debonding due to resin softening became more pronounced with increasing temperature and could be used to explain the degradation of FML’s tensile properties.

(5) Digital imaging technology clearly observed FML tensile dynamics. The strain distribution map showed different states as the load increased. The stress distribution in the strain distribution map was concentrated around the circular holes. As the number of holes increased, the load-bearing capacity decreased sharply.

## Figures and Tables

**Table 1:** Mechanical property parameters of BF/EP prepregs.

Mechanical Property	Parameters	Mechanical Property	Parameters
$E_{11}/\mathrm{GPa}$	28.5	$S_{23}/\mathrm{GPa}$	41.38
$E_{22}/\mathrm{GPa}$	6.4	$X_{T}/\mathrm{MPa}$	500
$E_{33}/\mathrm{GPa}$	6.4	$Y_{T}=Z_{T}/\mathrm{MPa}$	6.5
$G_{12}=G_{13}/\mathrm{GPa}$	2.5	$Y_{C}=Z_{C}/\mathrm{MPa}$	60
$G_{23}/\mathrm{GPa}$	1.3	$v_{12}=v_{13}$	0.3
$S_{12}=S_{13}/\mathrm{GPa}$	41.38	$v_{23}$	0.3

**Table 2:** 2024-T3 aluminum alloy mechanical property parameters.

ρ	2700 kg/m^3^	Yield Strength	292 MPa	Fracture Energy	10,200 J/m^2^	
*E*	72 GPa	Poisson’s Ration	0.33	Fracture Strain	0.15	
**Isotropic Hardening Data**						
stress (MPa)	292	300	313	322	335	342
strain (%)	0	0.098	0.29	0.47	0.78	0.97
stress (MPa)	354	360	376	392	406	428
strain (%)	1.47	1.65	2.48	3.49	4.82	7.18

**Table 3:** 2024-T3 aluminum alloy material parameters.

Johnson–Cook Parameterizations of an Isomorphic Model				
*A*	*B*	*C*	*n*	*m*
369	684	0.001	0.73	2.75
Crack model parameters				
*D*_1_	*D*_2_	*D*_3_	*D*_4_	*D*_5_
0.112	0.123	−1.5	0.007	0

**Table 4:** Material parameters of the cohesive layer elements.

*E* (GPa)			*t*^0^ (MPa)			*G_c_* (N/mm)			Density (kg/mm^3^)	Final Temperature
$E_{\mathrm{nn}}$	$E_{\mathrm{ss}}$	$E_{\mathrm{tt}}$	$\sigma_{n}^{0}$	$\sigma_{s}^{0}$	$\sigma_{t}^{0}$	$G_{\mathrm{IC}}$	$G_{\mathrm{IIC}}$	$G_{\mathrm{IIIC}}$	$\rho_{c}$	T
2	0.75	0.75	65	38	38	2	4	4	920	180

**Table 5:** Ultimate tensile stress at different temperatures.

Cases	Sequence	Ultimate Tensile Stress (MPa)	Ultimate Tensile Stress (MPa)	Ultimate Tensile Stress (MPa)	Ultimate Tensile Stress (MPa)
		FMLs (single hole)	FMLs (two hole)	FMLs (four hole)	FMLs (six hole)
30 °C	1	190.78	177.77	163.60	149.34
	2	187.17	170.92	169.42	147.85
	3	182.33	182.41	162.19	148.84
	Average value	186.76	177.03	165.07	148.68
80 °C	1	199.57	181.62	171.81	166.77
	2	193.44	185.36	180.10	171.59
	3	204.46	182.09	174.80	169.29
	Average value	199.16	183.02	175.57	169.21
130 °C	1	178.75	129.18	152.09	128.71
	2	181.03	128.76	155.11	128.90
	3	172.98	128.01	159.90	125.54
	Average value	177.59	128.65	155.70	127.72
180 °C	1	160.37	136.70	103.23	93.97
	2	164.97	129.56	103.10	96.66
	3	168.45	139.51	102.37	92.90
	Average value	164.60	135.26	102.9	94.51

## Data Availability

The original contributions presented in the study are included in the article, further inquiries can be directed to the corresponding author.
